# The potential utility of an augmented data collection approach in understanding the journey to care of pregnant women for maternal and perinatal death surveillance and response

**DOI:** 10.12688/f1000research.123210.3

**Published:** 2022-09-23

**Authors:** Aduragbemi Banke-Thomas

**Affiliations:** 1 School of Human Sciences, University of Greenwich, London, SE10 9LS, UK; 2 Maternal and Reproductive Health Research Collective, Lagos, Nigeria

**Keywords:** Maternal mortality, Perinatal mortality, Maternal and Perinatal Death Surveillance and Response, Audit, Emergency obstetric care, Travel, Access to healthcare, Nigeria

## Abstract

**Background:** The Maternal and Perinatal Death Surveillance and Response (MPDSR) proposed by the World Health Organization recognises the importance for health systems to understand the reasons underpinning the death of a pregnant woman or her newborn as an essential first step in preventing future similar deaths. Data for the surveillance component of the MPDSR process are typically collected from health facility sources and post-mortem interviews with affected families, though it may be traumatising to them. This brief report aimed to assess the potential utility of an augmented data collection method for mapping journeys of maternal and perinatal deaths, which does not require sourcing additional information from grieving family members.

**Methods:** A descriptive analysis of maternal and perinatal deaths that occurred across 24 public hospitals in Lagos State, Nigeria, between 1 ^st^ November 2018 and 30 ^th^ October 2019 was conducted. Data on their demographic, obstetric history and complication at presentation, travel to the hospital, and mode of birth were extracted from their hospital records. The extracted travel data was exported to Google Maps, where driving distance and travel time to the hospital for the period of the day of travel were also extracted.

**Results:** Of the 182 maternal deaths, most presented during the week (80.8%), travelled 5-10 km (30.6%) and 10-29 minutes (46.9%), and travelled to the nearest hospital to their places of residence (70.9%). Of the 442 pregnant women who had perinatal deaths, most presented during the week (78.5%), travelled <5 km (26.9%) and 10-29 minutes (38.0%). For both, the least reported travel data was the mode of travel used to care (>90.0%) and the period of the day they travelled (approximately 30.0%).

**Conclusion:** An augmented data collection approach that includes accurate and complete travel data and closer-to-reality estimates of travel time and distance can be beneficial for MPDSR purposes.

## Introduction

Approximately 300,000 maternal deaths occur annually because of complications related to pregnancy and childbirth. These complications include abortion, pre-eclampsia/eclampsia, ante- or post-partum haemorrhage, and sepsis. ^1^ In addition, these complications also increase the chance of pregnant women having babies born dead or dying within the first week of life (perinatal deaths). It is estimated that about three million perinatal deaths occur every year. ^2^^,^^3^ Between 97% and 99% of these deaths occur in low- and middle-income countries (LMICs). ^1^^–^^3^ To minimise the risk of maternal and perinatal deaths, pregnant women need to be able to promptly access emergency obstetric care (EmOC) provided by skilled health personnel. ^4^^,^^5^ However, before pregnant women can access EmOC, they must first decide to seek the care, travel to a health facility with the capacity to provide EmOC and, upon arrival at the health facility, have a skilled health personnel who can actually provide the needed care or promptly refer them. ^6^ Travel time and distance to care may lead to maternal or perinatal deaths. ^7^^–^^10^

There is a global consensus that understanding the reasons underpinning the death of a pregnant woman or her unborn child is an important first step in forestalling future similar deaths. To reach this understanding, in addition to being able to label the obstetric complication that led to the death(s), it is crucial to capture the pregnant woman’s personal story to care and the precise circumstances around her death or that of her unborn child. To be comprehensive and useful for action, the story needs to capture the narrative and establish any obstacles that prevented the woman from accessing prompt care. In 2021, the World Health Organization (WHO) and partners launched the Maternal and Perinatal Death Surveillance and Response (MPDSR) to investigate maternal and perinatal deaths and act based on the findings. ^11^ This new guide builds on two previous guides that focused on capturing the story of the mother and the newborn separately. ^12^^,^^13^

As per the WHO MPDSR guide, data from the admission and discharge register, labour and childbirth ward register, and theatre or minor surgery record books will be helpful. In addition, patient records, including case notes, referral notes, postoperative notes, and laboratory results are deemed to contain relevant information to reflect the personal stories of women. ^11^ However, while patient records have copious detail to understand factors that might have contributed to delays after the woman arrived at the health facility, they typically contain minimal information on the journey she travelled to care. ^14^^,^^15^ The WHO recommends that though it is more difficult to obtain, such additional information could be sourced from the woman’s family. ^11^ In practice, this might mean conducting post-mortem interviews for MPDSR purposes, as in Indonesia. ^16^ However, the woman’s family are not always in the frame of mind to provide, and neither are the skilled health personnel to collect the necessary information when a death has occurred. ^17^^,^^18^ Other challenges, including additional workload for skilled health personnel, have been mentioned in the literature. ^19^ Indeed, issues related to travel to care are rarely specifically flagged as contributory factors to maternal or perinatal deaths reported in MPDSR audits conducted in LMICs. ^20^ The objective of this brief report was to assess the potential utility of an augmented data collection method that uses data already collected in patient case notes to map journeys of maternal and perinatal deaths in a navigation software without requiring any additional information from family members.

## Methods

### Ethical approval

Ethical approval for this study was obtained from the Human Research and Ethics Committee of Lagos University Teaching Hospital (ADM/DCST/HREC/APP/2880) and the Health Research and Ethics Committee of Lagos State University Teaching Hospital (LREC/06/10/1226). This study was conducted with secondary data from hospital records with permission from the Ministry of Health to access these records. There was no direct interaction with patients at any point in time. The risk of identifying pregnant women in the study was substantially reduced by not collecting identifiers such as names and specific street numbers.

### Study design

This descriptive study was conducted across all 24 public hospitals in Lagos State, Nigeria, that provided EmOC. Lagos is a principally urban state located in the southwestern part of Nigeria with a total population of 26 million as of 2019. ^21^ For different reasons, including perceived higher concentration of skilled health personnel and equipment, availability of round-the-clock care, and in some instances ‘free’ or reduced fees, many pregnant women prefer to access EmOC in public hospitals. ^22^ Institutional maternal mortality ratios in Lagos public hospitals have been reported to range between 987 and 2,111 per 100,000 live births. Over a third of maternal deaths are attributed to a delayed presentation at health facilities. ^23^

For this study, pregnant women who presented in the emergency room of the different public hospitals between 1 ^st^ November 2018 and 30 ^th^ October 2019 were identified. The sample for this brief report was limited to those who resulted in maternal deaths or had perinatal deaths. Amongst these women, data on demographic characteristics, obstetric history, travel to the hospital, obstetric complication (as defined in the WHO’s Monitoring EmOC guidelines), ^5^ and mode of birth were extracted.

Based on the travel data extracted from the patient records, additional data were collected to estimate the driving distance (in kilometres (km)) and travel time (in minutes (mins)) of the pregnant women to the hospital using Google Maps (Alphabet Inc., Mountain View, California, US), which offers closer-to-reality estimates. ^24^ To map the journeys in Google Maps, the street name of women’s self-reported addresses and referral points were geo-referenced for each woman who had traceable journeys in the application. The ‘typical time of travel’ feature in Google Maps was used for the period of the day of travel for specific time slots (9.00 a.m., 3.00 p.m., 6.00 p.m., and 9.00 p.m. for morning, afternoon, evening, or night journeys, respectively), based on awareness of peak and non-peak travel periods in Lagos. ^25^ A check was subsequently conducted in Google Maps to ascertain whether there was an alternative public hospital closer to the pregnant woman’s self-reported address for the period of the day of travel to care.

For this study, maternal death was defined as “the death of a woman while pregnant or within 42 days of termination of pregnancy, irrespective of the duration and site of the pregnancy, from any cause related to or aggravated by the pregnancy or its management, but not from accidental or incidental causes”. ^26^ Perinatal death was defined as a foetal death occurring on or after 28-week gestation but before birth or neonatal death within seven days of life. ^3^ A perinatal death was recorded as long as a foetal death occurred even If the woman had multiple gestations (e.g. twins) and one baby survived.

A descriptive analysis of the socio-demographic, obstetric, and travel characteristics of the women who ended as maternal deaths or had perinatal deaths was conducted. The data was disaggregated by referral status. Analysis was conducted using Stata SE version 16.1 (StataCorp, College Station, Texas, USA).

## Results

In all, there were 182 intra-facility maternal deaths amongst pregnant women who presented in the emergency rooms of the public hospitals during the study period. These maternal deaths included 140 (76.9%) pregnant women who travelled directly to hospitals where they received EmOC, and 42 (23.1%) were referred. Amongst all maternal deaths, the majority were pregnant women who were aged 20-34 years (68.1%), married (86.3%), self-employed as petty traders (44.04%), and had attained a secondary level of education (37.4%). In terms of obstetric history, most maternal deaths were pregnant women with no complication in any of their previous pregnancies (93.4%) and multiparous at presentation (42.9%). For their index pregnancy, most maternal deaths were pregnant women who were un-booked (94.0%), had singleton pregnancies (98.9%), and presented with spontaneous abortion (40.1%). Regarding travel to care, most maternal deaths were pregnant women who presented during the week (80.8%), travelled 5-10 km (30.6%) and 10-29 mins (46.9%). Journeys of 4.9% of women who ended as maternal deaths could not be mapped. Most travelled to the nearest hospital to their places of residence (70.9%). Most of those referred before they died initially presented at a primary health centre (40.5%). It was not possible to extract data on what period of the day they travelled (29.7%) or what mode of travel they used to care (92.9%) for most women who ended as maternal deaths, as these were not reported in the patient records [ Table 1].

**Table 1.** Socio-demographics, obstetric history, characteristics of index pregnancy, and travel to care for pregnant women who ended in maternal deaths.

Characteristics	Number of deaths ([%] n=182)	Referred ([%] n=42)	Not referred ([%] n=140)
Age			
12-19	10 (5·5)	2 (4·8)	8 (5·7)
20-34	124 (68·1)	30 (71·4)	94 (67·1)
35-60	48 (26·4)	10 (23·8)	38 (27·2)
Marital status			
Single	25 (13·7)	6 (14·3)	19 (13·6)
Married	157 (86·3)	36 (85·7)	121 (86·4)
Education level attained			
No formal education	18 (9.9)	12 (28.6)	6 (4.3)
Primary	9 (4.9)	4 (9.5)	5 (3.6)
Secondary	68 (37.4)	8 (19.1)	60 (42.8)
Tertiary	25 (13.7)	4 (9.5)	21 (15.0)
Not recorded	62 (34.1)	14 (33.3)	48 (34.3)
Employment status			
Unemployed/Housewife	33 (18·1)	11 (26·2)	22 (15·7)
Student	17 (9·3)	2 (4·8)	15 (10·7)
Self-employed (Petty-trader)	80 (44·0)	20 (4·8)	60 (42·9)
Self-employed (Mid-high business)	17 (9·3)	3 (7·1)	14 (10·0)
Employed	35 (19·2)	6 (14·3)	29 (20·7)
Obstetric complications in a previous pregnancy			
Yes	12 (6·6)	3 (7·1)	39 (92·9)
No	170 (93·4)	3,448 (82·5)	131 (93·6)
Parity			
Nulliparous (0)	59 (32.4)	16 (38.1)	43 (30.7)
Primiparous (1)	40 (22.0)	8 (19.0)	32 (22.9)
Multiparous (2-4)	78 (42.9)	18 (42.9)	60 (42.9)
Grand-multiparous (5 or more)	5 (2.8)	0 (0.0)	5 (3.6)
Number of gestations			
Singleton	180 (98·9)	40 (100·0)	140 (98·6)
Multiple	2 (1·1)	0 (0·0)	2 (1·4)
Booking status			
Booked	11 (6·0)	1 (2·4)	10 (7·1)
Un-booked	171 (94·0)	41 (97·6)	130 (92·9)
Obstetric complication			
Foetal complication	6 (3·3)	1 (2·4)	5 (3·6)
Obstructed labour	9 (5·0)	5 (11·8)	4 (2·9)
Haemorrhage	26 (14·3)	8 (19·1)	18 (12·9)
Pre-eclampsia/eclampsia	31 (17·0)	8 (19·1)	23 (16·4)
Sepsis	6 (3·3)	2 (4·7)	4 (2·9)
Abortion	73 (40·1)	14 (33·4)	59 (42.2)
Ectopic pregnancy	22 (12·1)	3 (7·1)	19 (13.6)
Others	9 (5·0)	1 (2·4)	9 (6·4)
Weekend travel to facility			
Yes	35 (19·2)	10 (23·8)	25 (17·9)
No	147 (80·8)	32 (76·2)	115 (82·1)
Period of the day of travel to the facility			
Morning	32 (17·6)	9 (21·4)	23 (16·4)
Afternoon	38 (20·9)	11 (26·2)	27 (19·3)
Evening	37 (20·3)	4 (9·5)	33 (23·6)
Night	21 (11·5)	5 (11·9)	16 (11·4)
Could not tell	54 (29.7)	13 (31.0)	41 (29.3)
Initial point of care for those referred			
Another public hospital	4 (9·5)	4 (9·5)	-
Private hospital	10 (23·8)	10 (23·8)	-
Private clinic	2 (4·8)	2 (4·8)	-
Primary health centre	17 (40·5)	17 (40·5)	-
Traditional birth attendant	8 (19·1)	8 (19·1)	-
Nursing/maternity home	0 (0·0)	0 (0·0)	-
Non-formal referral	1 (2·4)	1 (2·4)	-
Distance to the facility of delivery			
Within 5 km	47 (25.8)	10 (23.8)	37 (26.4)
5-10 km	52 (28.6)	10 (23.8)	42 (30.0)
>10-15 km	31 (17.0)	7 (16.7)	24 (17.1)
>15-25 km	20 (11.0)	7 (16.7)	13 (9.3)
>25-35 km	11 (6.1)	4 (9.5)	7 (5.0)
>35 km	12 (6.6)	4 (9.5)	8 (5.7)
Could not trace journey	9 (4.9)	0 (0.0)	9 (6.4)
Time to the facility of delivery			
0-9 minutes	17 (9.4)	2 (4.8)	15 (10.7)
10-29 minutes	84 (46.2)	18 (42.9)	66 (47.1)
30-59 minutes	45 (24.7)	13 (30.9)	32 (22.9)
60-119 minutes	20 (11.0)	8 (19.0)	12 (8.6)
120-480 minutes	7 (3.8)	1 (2.4)	6 (4.3)
Could not trace journey	9 (4.9)	0 (0.0)	9 (6.4)
Proximity of alternative hospital			
No	129 (70.9)	30 (71.4)	99 (70.7)
Yes	44 (24.2)	12 (28.6)	32 (22.9)
Could not trace journey	9 (4.9)	0 (0.0)	9 (6.4)
Mode of travel			
Private car	3 (1.7)	1 (2.4)	2 (1.4)
Taxi	1 (0.5)	0 (0.0)	1 (0.7)
Tricycle	2 (1.1)	0 (0.0)	2 (1.4)
Motorcycle	1 (0.5)	0 (0.0)	1 (0.7)
Bus	2 (1.1)	1 (2.4)	1 (0.7)
Ambulance	4 (2.2)	4 (9.5)	0 (0.0)
Not recorded	169 (92.9)	36 (85.7)	133 (95.1)

There were 442 intra-facility perinatal deaths amongst pregnant women who presented in public hospitals requiring EmOC during the study period, including 269 (60.9%) who travelled directly to the hospital where they received EmOC and 173 (39.1%) referred. Most pregnant women who had perinatal deaths were aged 20-34 years (67.2%), married (94.3%), and self-employed as petty traders (43.0%). Most did not have the level of education attained reported in their case notes (52.3%). In terms of obstetric history, most perinatal deaths were delivered by pregnant women who did not have a complication in a previous pregnancy (93.4%) and were multiparous at presentation (43.4%). For the index pregnancy, most perinatal deaths were by un-booked mothers (81.2%) and were singleton pregnancies (96.8%). Regarding travel, most perinatal deaths were delivered by pregnant women who presented during the week (78.5%), travelled <5 km (26.9%) and 10-29 minutes (38.0%). Journeys of 4.8% of women with perinatal deaths could not be mapped. Most travelled to the nearest hospital to their places of residence (70.9%). Most of those referred before they died initially presented at a primary health centre (37.3%). For most pregnant who ended with a perinatal death, it was not possible to extract data on the period of the day they travelled (34.6%) or the mode of travel used to care (98.9%) as these were not reported in the patient records. Most foetuses that ended as perinatal deaths were delivered via spontaneous vaginal birth (56.6%) [ Table 2].

**Table 2.** Socio-demographics, obstetric history, characteristics of index pregnancy, and travel history of pregnant women who delivered stillbirths.

Characteristics	Number of deaths ([%] n=442)	Referred ([%] n=173)	Not referred ([%] n=269)
Age			
12-19	11 (2·5)	6 (3·5)	5 (1·9)
20-34	297 (67·2)	119 (68·8)	178 (66·2)
35-60	134 (30·3)	48 (27·7)	86 (31·9)
Marital status			
Single	25 (5·7)	6 (3·5)	19 (7·1)
Married	417 (94·3)	167 (96·5)	250 (92·9)
Education level attained			
No formal education	108 (24.4)	62 (35.8)	46 (17.1)
Primary	11 (2.5)	7 (4.0)	4 (1.5)
Secondary	61 (13.8)	27 (15.6)	34 (12.6)
Tertiary	31 (7.0)	11 (6.4)	20 (7.4)
Not recorded	231 (52.3)	66 (38.2)	165 (61.3)
Employment status			
Unemployed/Housewife	98 (22·2)	41 (23·7)	57 (21·2)
Student	21 (4·7)	7 (4·1)	14 (5·2)
Self-employed (Petty-trader)	190 (43·0)	81 (46·8)	109 (40·5)
Self-employed (Mid-high business)	64 (14·5)	20 (11·6)	44 (16·4)
Employed	69 (15·6)	24 (13·9)	45 (16·7)
Obstetric complications in a previous pregnancy			
Yes	84 (19·0)	30 (17·3)	54 (20·1)
No	358 (81·0)	143 (82·7)	215 (79·9)
Parity			
Nulliparous (0)	119 (26.9)	43 (24.9)	78 (28.3)
Primiparous (1)	113 (25.6)	47 (27.2)	66 (24.5)
Multiparous (2-4)	192 (43.4)	80 (46.2)	112 (41.6)
Grand-multiparous (5 or more)	18 (4.1)	3 (1.7)	15 (5.6)
Number of gestations			
Singleton	428 (96·8)	168 (97·1)	260 (96·7)
Multiple	14 (3·2)	5 (2·9)	9 (3·3)
Booking status			
Booked	83 (18·8)	9 (5·2)	74 (27·5)
Un-booked	359 (81·2)	164 (94·8)	195 (72·5)
Weekend travel to facility			
Yes	95 (21·5)	34 (19·6)	61 (22·7)
No	347 (78·5)	139 (80·4)	208 (77·3)
Period of the day of travel to the facility			
Morning	103 (23·3)	32 (18·5)	71 (26·4)
Afternoon	71 (16·1)	25 (14·5)	46 (17·1)
Evening	65 (14·7)	19 (11·0)	46 (17·1)
Night	50 (11·3)	11 (11·5)	30 (11·1)
Could not tell	1553 (34.6)	77 (44.5)	76 (28.3)
Initial point of care for those referred			
Another public hospital	24 (13·8)	24 (13·8)	-
Private hospital	46 (26·4)	46 (26·4)	-
Private clinic	2 (1·6)	2 (1·6)	-
Primary health centre	65 (37·3)	65 (37·3)	-
Traditional birth attendant	30 (17·2)	30 (17·2)	-
Nursing/maternity home	4 (0·5)	4 (0·5)	-
Non-formal referral	16 (2·2)	16 (2·2)	-
Distance to the facility of delivery			
Within 5 km	119 (26.9)	35 (20.2)	84 (31.2)
5-10 km	110 (24.9)	35 (20.2)	75 (27.9)
>10-15 km	53 (12.0)	29 (16.8)	24 (8.9)
>15-25 km	61 (13.8)	29 (16.8)	32 (11.9)
>25-35 km	23 (5.2)	10 (5.8)	13 (4.5)
>35 km	55 (12.4)	35 (20.2)	20 (7.4)
Could not trace journey	21 (4.8)	0 (0.0)	21 (0.4)
Time to the facility of delivery			
0-9 minutes	41 (9.3)	12 (6.9)	29 (10.8)
10-29 minutes	168 (38.0)	53 (30.6)	115 (42.8)
30-59 minutes	108 (24.4)	49 (28.3)	59 (21.9)
60-119 minutes	81 (18.3)	42 (24.3)	39 (14.5)
120-480 minutes	23 (5.2)	17 (9.8)	6 (2.2)
Could not trace journey	21 (4.8)	0 (0.0)	21 (7.8)
Proximity of alternative hospital			
No	229 (70.9)	134 (77.5)	95 (35.3)
Yes	192 (24.2)	39 (22.5)	153 (56.9)
Could not trace journey	21 (4.9)	0 (0.0)	21 (7.8)
Mode of travel			
Private car	2 (0.5)	2 (1.4)	1 (0.4)
Taxi	1 (0.2)	1 (0.7)	1 (0.4)
Tricycle	0 (0.0)	2 (1.4)	0 (0.0)
Motorcycle	1 (0.2)	1 (0.7)	1 (0.4)
Bus	1 (0.2)	1 (0.7)	0 (0.0)
Ambulance	0 (0.0)	0 (0.0)	0 (0.0)
Not recorded	437 (98.9)	171 (98.8)	266 (98.8)
Mode of birth			
Spontaneous vaginal birth	250 (56·6)	85 (49·1)	165 (61·3)
Assisted vaginal birth	30 (6·8)	9 (5·2)	21 (7·8)
Caesarean birth	162 (36·6)	79 (45·7)	83 (30·9)

## Discussion

This brief report showed that for MPDSR, patient records are useful in capturing the personal stories relating to travel to care which might have contributed to maternal and perinatal deaths. Data on the day of travel and whether this is a weekday or weekend, which is important because of varying availability of transport options and degrees of traffic, ^14^ were available in all cases. Having data on specific travel dates will be helpful in contextualising strikes, periods of petrol scarcity, lockdowns, protests, road blockages etc. which may all affect travel. Data on some relevant socio-demographic and all obstetric history, which will be helpful for efforts in building travel-relevant context of the maternal and perinatal deaths, were reported. However, data that will allow full characterisation of the travel to care are not always completely reported in case notes. As per evidence gathered from this study, questions relating to the period of the day of travel to the facility and mode of transport are only minimally recorded. In addition, there were cases of incomplete, wrong, or difficult-to-read addresses, which made it impossible to locate residential addresses. For those referred, though the type of referral facility was reported in many instances (for example, by simply writing ‘private clinic’), it was not always possible to map the actual location of the referral facilities. The utility of the travel data when complete and reflective of the travel to care was further improved when complementary travel data, including travel time and distance, were subsequently collected using a web-based navigation application (Google Maps). This study showed that data was more detailed for maternal deaths compared to perinatal deaths.

These study findings have several implications for practice and policy, especially as issues related to travel to care are seldomly flagged in MPDSR audits conducted in LMICs. ^20^ First, as with the recognised need for complete and accurate information on the circumstance and management of pregnant women and their newborns at all levels, ^27^ skilled health personnel need to be trained and encouraged to collect detailed and accurate travel history of pregnant women at the point of presentation, with a guaranty of no blame at audit even if there was a delay in a referral or organising an ambulance for onward travel. ^17^^,^^28^ These efforts need to include verification of points of origin from which the woman came to care, which may be their home or anywhere else in the community. In instances where the points of origin are difficult to establish, a nearby popular structure (for example, ‘beside the stadium’) should be inputted as a proxy. Indeed, this process of address localisation will be easier with electronic health information systems. However, challenges related to the cost of implementing and maintaining such systems have been raised. ^29^ The alternative to this, which is also the status quo in many LMIC health systems, involves using hand-written paper-based platforms. However, this is prone to errors. ^29^ As was observed in this study, errors related to accurate reporting of patient addresses limit the utility of the data for assessing delays that might have contributed to maternal or perinatal deaths. In deciding the health information management system to implement, the efficiency, accuracy, data safe-keeping, and decision-making gains that come with electronic systems need to be considered as they may guarantee value for money for such investments. ^29^^–^^33^

The augmented data collection approach used for this research yielded additional information that would otherwise not have been available. Beyond understanding the journey to care preceding the death, insights garnered from this augmented data collection approach can help provide the more robust evidence to support the planning of EmOC services. ^32^^,^^34^ This approach of leveraging technology to estimate travel time and distance has been shown to offer closer-to-reality estimates, especially in urban areas. ^24^ Indeed, there might still be a case for collecting additional information from family members. For example, to establish if there were notably worse traffic conditions beyond the ‘typical travel time’ reported by Google Maps or a motor vehicle breakdown that will not be captured in Google Maps in any case. An enquiry might also still be required to establish circumstances which might have contributed to delays in the decision to seek care. However, these supplementary enquiries risk re-traumatising relatives after the death of their loved ones. The proposed augmented data collection approach in this study will reduce the number of families that need to be engaged and could potentially improve the efficiency of MPDSR committees. In instances in which an enquiry is still warranted, the augmented data collection proposed in this report could serve the purpose of data triangulation.

There are some limitations to consider in interpreting the findings of this study. First, though Google Maps has been shown to provide closer-to-reality estimates of travel time and distance in urban settings like Lagos, its applicability in rural settings remains questionable. ^24^ Second, an assumption was made that all cases used a motor vehicle to reach the health facility where they received care. While this may not always be the case, available evidence shows that nine in 10 pregnant women in emergency situations travel to care in a four-wheel vehicle in Nigeria. ^35^ Third, the study was conducted with retrospective health facility data. While this provided an actual case study in an unaltered environment, it did not allow exploration of the full potential of this augmented approach if instituted, building on complete and accurate data that could have been realised if the study had been conducted prospectively. Future prospective research needs to be undertaken, and the utility of this augmented data collection approach needs to be assessed from the perspective of MPDSR committee members.

## Conclusions

In conclusion, while not the magic bullet, for MPDSR purposes, an augmented data collection approach that includes accurate and complete travel data collection and closer-to-reality estimates of travel time and distance can improve the understanding of travel experiences of pregnant women and their new-borns to care. The usefulness of information already collected in patient records can be significantly improved if more thorough travel to care history that captures the period of the day of commencement of travel to the health facility, mode of travel, condition of road during travel, referral points, time of referral, major incidents that might have affected or delayed travel, and arrival time at the health facility are taken when the pregnant woman presents in an emergency.

## Data availability statement

### Underlying data

Figshare: Intra-facility_maternal_deaths_Lagos_2018-2019.csv. https://doi.org/10.6084/m9.figshare.20098148.v1. ^36^

This project contains the following underlying data:

• Intra-facility_maternal_deaths_Lagos_2018-2019.csv, (Anonymised data on maternal deaths analysed in this study).
• Intra-facility_perinatal_deaths_Lagos_2018-2019.csv, (Anonymised data on perinatal deaths analysed in this study).

Data are available under the terms of the Creative Commons Attribution 4.0 International license (CC-BY 4.0).
