# Supplementary material for: The potential utility of an augmented data collection approach in understanding the journey to care of pregnant women for maternal and perinatal death surveillance and response
Source: F1000Res. 2022 Sep 23;11:739. Originally published 2022 Jul 4. [Version 3] doi: 10.12688/f1000research.123210.3 (PMC9475203; doi:10.12688/f1000research.123210.3)
Supplement: Peer review history

# Peer review: Reviewer response for version 2

**DOI:** 10.5256/f1000research.137317.r149755

**Published:** 2022-09-14

**Authors:** Prestige Tatenda Makanga

**Affiliations:** 1 Surveying and Geomatics Department, Faculty of Science and Technology, Midlands State University, Gweru, Zimbabwe

I think all the issues raised have been adequately addressed.

Is the work clearly and accurately presented and does it cite the current literature?

Yes

If applicable, is the statistical analysis and its interpretation appropriate?

Yes

Are all the source data underlying the results available to ensure full reproducibility?

Yes

Is the study design appropriate and is the work technically sound?

Yes

Are the conclusions drawn adequately supported by the results?

Yes

Are sufficient details of methods and analysis provided to allow replication by others?

Yes

Reviewer Expertise:

Spatial Epidemiology, Health Geography

I confirm that I have read this submission and believe that I have an appropriate level of expertise to confirm that it is of an acceptable scientific standard.

# Peer review: Reviewer response for version 2

**DOI:** 10.5256/f1000research.137317.r149754

**Published:** 2022-09-09

**Authors:** Tope Olubodun

**Affiliations:** 1 Department of Community Medicine and Primary Care, Federal Medical Center, Abeokuta, Ogun State, Nigeria

Thank you for the revisions which have been well done. As the change has been made in the methods to reflect that the study was conducted in 24 public hospitals that provide emergency obstetric services, the abstract should also reflect that the study was done **in** 24 public hospitals in Lagos, rather than **all** 24 public hospitals in Lagos.

Is the work clearly and accurately presented and does it cite the current literature?

Yes

If applicable, is the statistical analysis and its interpretation appropriate?

Yes

Are all the source data underlying the results available to ensure full reproducibility?

Yes

Is the study design appropriate and is the work technically sound?

Yes

Are the conclusions drawn adequately supported by the results?

Yes

Are sufficient details of methods and analysis provided to allow replication by others?

Yes

Reviewer Expertise:

Public Health, Reproductive Health.

I confirm that I have read this submission and believe that I have an appropriate level of expertise to confirm that it is of an acceptable scientific standard.

# Peer review: Reviewer response for version 1

**DOI:** 10.5256/f1000research.135294.r143182

**Published:** 2022-08-04

**Authors:** Prestige Tatenda Makanga

**Affiliations:** 1 Surveying and Geomatics Department, Faculty of Science and Technology, Midlands State University, Gweru, Zimbabwe

This is an important report that furthers the conversation on ways to understand pathways to deaths for women due to complications of pregnancy and child birth. It's timely considering the 2021 development in maternal and perinatal deaths surveillance. It addresses the limitations of current methods of gathering data, that somewhat compromise privacy, still collecting limited and subjective information. That said, there are a few minor issues that the author needs to address before the report can be published.

1. The author needs to describe or define what they mean by an augmented approach early in the article. It becomes clear as one reads, but a definition would help.
2. Though demographic characteristics and obstetric history serve as important information that gives context to any study of this kind, it is unclear how this is valuable for this specific report seeing that the author is emphasizing collecting data related to the women's travel. At the least, the author could minimize this information and make the tables smaller.
3. Why is it necessary to differentiate weekdays and weekends? The author should explain and also reflect on this in the discussion.
4. "self-employed as petty traders (37.4%)" in results should be 44%.
5. "In terms of obstetric history, most maternal deaths were pregnant women with complications in a previous pregnancy (93.4%)" in results. From the Table, it is "NO" with a score of 93.4% implying that 6.6 did not have complications in previous pregnancies'. Do check if my interpretation is correct.
6. It would be appropriate for the author to address in greater detail the impact of the 42 that were referred on modelling the travel times, since the current approach estimates travel directly to the facility where the woman died. Also since mode was not known for 93% of the record, what would be the implication on assuming that everyone was driven?
7. Add period of travel to table 1. 
8. In the discussion or conclusion, I suggest that the author suggest specific fields that could be added to current data collection instruments to aid the augmentation that they are suggesting.

Is the work clearly and accurately presented and does it cite the current literature?

Yes

If applicable, is the statistical analysis and its interpretation appropriate?

Yes

Are all the source data underlying the results available to ensure full reproducibility?

Yes

Is the study design appropriate and is the work technically sound?

Yes

Are the conclusions drawn adequately supported by the results?

Yes

Are sufficient details of methods and analysis provided to allow replication by others?

Yes

Reviewer Expertise:

Spatial Epidemiology, Health Geography

I confirm that I have read this submission and believe that I have an appropriate level of expertise to confirm that it is of an acceptable scientific standard, however I have significant reservations, as outlined above.

# Response

**Published:** 2022-08-10

**Authors:** Aduragbemi Banke-Thomas

**Affiliations:** University of Greenwich, UK

*"This is an important report that furthers the conversation on ways to understand pathways to death for women due to complications of pregnancy and childbirth. It's timely considering the 2021 development in maternal and perinatal deaths surveillance. It addresses the limitations of current methods of gathering data, that somewhat compromise privacy, while still collecting limited and subjective information. That said, there are a few minor issues that the author needs to address before the report can be published."*

**Response**: Thank you for your thorough review which has really helped to strengthen the report and improve its clarity. Deeply appreciated.

**Comment 1:** *"The author needs to describe or define what they mean by an augmented approach early in the article. It becomes clear as one reads, but a definition would help."*

**Response**: Thank you for this comment. The objective has been modified to include a phrase that describes the proposed augmented approach early on. The objective now reads, "...to assess the potential utility of an augmented data collection method that uses data already collected in patient case notes to map journeys of maternal and perinatal deaths in navigation software without requiring additional information from family members".

**Comment 2**: *"Though demographic characteristics and obstetric history serve as important information that gives context to any study of this kind, it is unclear how this is valuable for this specific report seeing that the author is emphasizing collecting data related to the women's travel. At the least, the author could minimize this information and make the tables smaller."*

**Response**: Both demographic and obstetric data are critical for understanding the personal stories as described by the World Health Organization and better understanding the context before the travel. This has been highlighted in the discussion, where I added "Data on some relevant socio-demographic and all obstetric history, which will be helpful for efforts in building travel-relevant context of the maternal and perinatal deaths, were reported".

**Comment 3:** *"Why is it necessary to differentiate weekdays and weekends? The author should explain and also reflect on this in the discussion."*

**Response**: Thank you for this comment. An explanation has been added to the discussion, writing, "Data on the day of travel and whether this is a weekday or weekend, which is important because of varying availability of transport options and degrees of traffic, ^14^ were available in all cases. Having data on specific travel dates will be helpful in contextualising strikes, periods of petrol scarcity, lockdowns, protests, road blockages etc. which may all affect travel".

**Comment 4**: *""self-employed as petty traders (37.4%)" in results should be 44%."*

**Response**: Thank you for picking this error. This has now been changed in the results.

**Comment 5**: *""In terms of obstetric history, most maternal deaths were pregnant women with complications in a previous pregnancy (93.4%)" in results. From the Table, it is "NO" with a score of 93.4% implying that 6.6 did not have complications in previous pregnancies'. Do check if my interpretation is correct."*

**Response**: Thank you for picking this error. This has now been changed in the results. The statement now reads, "In terms of obstetric history, most maternal deaths were pregnant women with no complication in their previous pregnancy (93.4%)"

**Comment 6**: *"It would be appropriate for the author to address in greater detail the impact of the 42 that were referred on modelling the travel times, since the current approach estimates travel directly to the facility where the woman died. Also since mode was not known for 93% of the record, what would be the implication on assuming that everyone was driven?"*

**Response**: As described in the methods, "To map the journeys in Google Maps, the street name of women’s self-reported addresses and referral points were geo-referenced for each woman who had traceable journeys in the application". The journey of those referred followed their actual path to care. A note on the limitation of assuming that all women were driven has been added as a limitation, writing, "Second, an assumption was made that all cases used a motor vehicle to reach the health facility where they received care. While this may not always be the case, available evidence shows that nine in 10 pregnant women in emergency situations travel to care in a four-wheel vehicle in Nigeria. ^35"^

**Comment 7**: *"Add period of travel to table 1."*

**Response**: This is in the table.

**Comment 8**: *"In the discussion or conclusion, I suggest that the author suggest specific fields that could be added to current data collection instruments to aid the augmentation that they are suggesting."*

**Response**: Thank you for this very important recommendation. The concluding statement now reads "The usefulness of information already collected in patient records can be significantly improved if more thorough travel to care history that captures the period of the day of commencement of travel to the health facility, mode of travel, condition of road during travel, referral points, time of referral, major incidents that might have affected or delayed travel, and arrival time at the health facility are taken when the pregnant woman presents in an emergency.

 Once again, thank you very much for your detailed review.

# Peer review: Reviewer response for version 1

**DOI:** 10.5256/f1000research.135294.r143184

**Published:** 2022-07-26

**Authors:** Tope Olubodun

**Affiliations:** 1 Department of Community Medicine and Primary Care, Federal Medical Center, Abeokuta, Ogun State, Nigeria

This paper explains how an augmented data collection approach that includes accurate and complete travel data collection and closer-to-reality estimates of travel time and distance can improve the practice of maternal and perinatal death surveillance and response. The paper has findings which can be important for practice. It is well written, passes the message across and is at the same time, concise.

 Suggestions for revisions are provided below:

**Abstract**

 Please state that the study was carried out across all 24 public hospitals in Lagos State, that provided EmOC (emergency obstetric care). It is important to state that those public hospitals that provided EmOC were used, as some other public hospitals may exist that do not provide EmOC.

**Methods**

 The correct name of the Lagos University Teaching Hospital ethics committee is Human Research and Ethics Committees of the Lagos University Teaching Hospital.

**Discussion**

 Please make it clearer, how this approach will reduce the number of families that need to be engaged (third paragraph).

Is the work clearly and accurately presented and does it cite the current literature?

Yes

If applicable, is the statistical analysis and its interpretation appropriate?

Yes

Are all the source data underlying the results available to ensure full reproducibility?

Yes

Is the study design appropriate and is the work technically sound?

Yes

Are the conclusions drawn adequately supported by the results?

Yes

Are sufficient details of methods and analysis provided to allow replication by others?

Yes

Reviewer Expertise:

Public Health, Reproductive Health.

I confirm that I have read this submission and believe that I have an appropriate level of expertise to confirm that it is of an acceptable scientific standard, however I have significant reservations, as outlined above.

# Response

**Published:** 2022-07-30

**Authors:** Aduragbemi Banke-Thomas

**Affiliations:** University of Greenwich, UK

This paper explains how an augmented data collection approach that includes accurate and complete travel data collection and closer-to-reality estimates of travel time and distance can improve the practice of maternal and perinatal death surveillance and response. The paper has findings which can be important for practice. It is well written, passes the message across and is at the same time, concise.

 Response: Thank you for your thorough review. Deeply appreciated.

 Suggestions for revisions are provided below:

**Abstract**

*"Please state that the study was carried out across all 24 public hospitals in Lagos State, that provided EmOC (emergency obstetric care). It is important to state that those public hospitals that provided EmOC were used, as some other public hospitals may exist that do not provide EmOC."*

**Response:** Thank you for this important point, which has now been effected and will be reflected in the updated version when it is published. The statement now reads "A descriptive analysis of maternal and perinatal deaths that occurred in 24 emergency obstetric care providing public hospitals in Lagos, Nigeria...".

**Methods**

*"The correct name of the Lagos University Teaching Hospital ethics committee is Human Research and Ethics Committees of the Lagos University Teaching Hospital."*

**Response:** This is true. However, the sentence here was reflecting the two research and ethics committees for both Lagos University Teaching Hospital and Lagos State University Teaching Hospital. This was to keep the text succinct. To avoid confusion, both have been separated. It now reads "Ethical approval for this study was obtained from the Human Research and Ethics Committee of Lagos University Teaching Hospital (ADM/DCST/HREC/APP/2880) and the Health Research and Ethics Committee of Lagos State University Teaching Hospital (LREC/06/10/1226)". This change will be reflected in the updated version when it is published.

**Discussion**

*"Please make it clearer, how this approach will reduce the number of families that need to be engaged (third paragraph)."*

**Response:** Thank you for this comment. Sections of this paragraph have been rephrased to improve clarity. It now reads "For example, to establish if there were notably worse traffic conditions beyond the ‘typical travel time’ reported by Google Maps or a motor vehicle breakdown that will not be captured in Google Maps in any case. An enquiry might also still be required to establish circumstances which might have contributed to delays in the decision to seek care. However, these supplementary enquiries risk re-traumatising relatives after the death of their loved ones. The proposed augmented data collection approach in this study will reduce the number of families that need to be engaged and could potentially improve the efficiency of MPDSR committees".
